# Clinical Features and Recurrence Risk of Human Papillomavirus–Related Pharyngeal Papilloma: A Retrospective Study

**DOI:** 10.1002/oto2.70244

**Published:** 2026-04-27

**Authors:** Ryohei Asai, Hiroumi Matsuzaki, Kiyoshi Makiyama, Takeshi Oshima

**Affiliations:** ^1^ Department of Otolaryngology–Head and Neck Surgery Nihon University Hospital Tokyo Japan

**Keywords:** human papillomavirus, koilocytosis, multiplicity, pharyngeal papilloma, recurrence

## Abstract

**Objective:**

The association between pharyngeal papilloma and human papillomavirus (HPV) remains unclear. Although previous studies have suggested that HPV involvement is unlikely, we encountered HPV‐positive cases, particularly in patients with multiple lesions, malignancy, or recurrence. This study aimed to clarify the relationship between HPV infection and clinicopathological features of pharyngeal papilloma, including recurrence and malignant transformation.

**Study Design:**

Retrospective study.

**Setting:**

Department of Otolaryngology–Head and Neck Surgery, Nihon University Hospital, a single tertiary referral center in Tokyo, Japan.

**Methods:**

We retrospectively reviewed HPV‐DNA test results and clinical data from 69 patients with pharyngeal papilloma who underwent surgical resection between August 2006 and September 2018. Koilocytosis was histologically assessed, and associations between HPV status and recurrence, multiplicity, and malignant transformation were analyzed.

**Results:**

Among 69 patients, 5 (7.2%) were HPV‐positive, including four (5.8%) low‐risk types (6, 11) and 1 (1.4%) high‐risk type (16). Koilocytosis was observed in 88% of cases. HPV‐positive cases had higher recurrence, multiplicity, and malignant transformation rates compared with HPV‐negative cases.

**Conclusion:**

HPV DNA was detected in 7.2% of cases. HPV‐positive papillomas showed higher recurrence and malignanct transformation rates. Detection of HPV DNA may serve as a prognostic factor, and koilocytosis in HPV‐negative cases may reflect prior transient HPV infection.

Pharyngeal papilloma is the most common benign pharyngeal tumor, and malignant transformation appears to be uncommon; management typically involves excision, although careful observation with follow‐up may be considered depending on symptoms, size, and clinical course. In contrast, laryngeal papillomas are known to be human papillomavirus (HPV)–related and may cause dysphonia or even malignant transformation, often requiring surgical treatment.^1^ HPV is involved in the pathogenesis of laryngeal papilloma, and laryngeal papillomas have been reported to cause severe dysphonia and even cancer, making surgical excision the standard of care. However, they are often difficult to treat, and the disease recurs easily in some cases, such as recurrent respiratory papillomatosis.

Recently, the incidence of HPV‐associated oropharyngeal cancer has been increasing. A Japanese multicenter study reported that HPV was detected in more than 50% of patients with oropharyngeal cancer,^2^ raising concerns regarding HPV infection in the pharynx. However, few studies have examined the association between pharyngeal papilloma and HPV infection, with findings being inconsistent and positivity rates ranging from 0% to 53.8%.^3^, ^4^, ^5^, ^6^ A previous Japanese study found no association between pharyngeal papilloma and HPV infection.^3^

Based on our clinical observations of HPV‐positive pharyngeal papillomas exhibiting recurrence, multifocality, and malignant transformation, we conducted this retrospective study to determine whether HPV infection is associated with more aggressive clinical behavior in pharyngeal papillomas. We hypothesized that HPV infection is associated with the occurrence of multiple lesions, recurrence, and malignant transformation, and that detection of HPV DNA in pharyngeal papillomas may help predict prognosis.

## Materials and Methods

### Participants

This retrospective study included 69 patients with pharyngeal papilloma who underwent surgical resection at Nihon University Hospital between August 2006 and September 2018. All cases were histologically confirmed as squamous cell papilloma. Patients with concurrent laryngeal lesions were excluded to focus specifically on pharyngeal disease. This study was approved by the Institutional Review Board of Nihon University Hospital (approval no. 20210401). Written informed consent was obtained from all patients.

### HPV‐DNA Testing

Two complementary HPV DNA tests were performed for all patients. The first test employed liquid‐phase hybridization using pharyngeal secretions obtained near the lesion to screen for high‐risk HPV types (16, 18, 31, 33, 35, 39, 45, 51, 56, 58, 59, 68) and low‐risk types (6, 11, 42, 43, 44).^7^ The second test employed consensus primer–directed polymerase chain reaction using tumor tissue samples for HPV typing.^8^

### Histological Assessment: Koilocytosis Evaluation

Koilocytosis is a cellular phenomenon characterized by perinuclear vacuolization, nuclear enlargement, and irregular nuclear contours in the middle and superficial layers of stratified squamous epithelium. It is considered a histopathological hallmark of papillomavirus infection.^9^ Orita et al^10^ reported a significant association between koilocytosis and HPV DNA positivity in laryngeal papillomas, supporting its use as a histopathological indicator of HPV involvement. In this study, koilocytosis was evaluated in 43 available cases by an institutional pathologist.

### Tumor Size

Tumor diameters were recorded for 27 cases with available pathology reports and categorized at 4‐mm intervals.

### Follow‐Up Assessment

Follow‐up data were retrospectively obtained from medical records. Many patients were referred back to their primary physicians after surgery, resulting in variable follow‐up durations. The median follow‐up period was 7 months (range, 0‐114 months); 8 patients (11.6%) had no follow‐up at our institution, and 17 (24.6%) were followed for 12 months or longer.

### Clinical Variables

The following clinical parameters were compared between HPV‐positive and HPV‐negative cases: recurrence, multiplicity, and malignant transformation. Recurrence was defined as the reappearance of lesions after complete excision or a history of previous resection. Multiplicity was defined as the presence of 2 or more papillomas within the pharynx. Malignant transformation was defined as the presence of histopathological features of invasion within a lesion otherwise diagnosed as squamous cell papilloma.

### Statistical Analysis

Categorical variables were analyzed using the chi‐square or Fisher exact test, and continuous variables were analyzed using the Mann‐Whitney *U* test. A *P* < .05 was considered statistically significant. All statistical analyses were performed using GraphPad Prism version 8.4 (GraphPad Software).

## Results

### Patient Characteristics

The cohort included 39 men and 30 women aged 15 to 81 years (median age, 48 years). Clinical characteristics are summarized in Table 1. 10 patients had multiple lesions, 2 had recurrent disease, and 2 showed malignant transformation. Lesions were most frequently confined to the oropharynx (46/69, 66.7%), followed by the hypopharynx (19/69, 27.5%) and nasopharynx (1/69, 1.4%). Three patients (4.3%) had lesions involving more than 1 pharyngeal region. Within the oropharynx, the superior wall (soft palate/uvula) was the most common site.

**Table 1:** Clinical Characteristics of the Study Group

Characteristic	Papilloma group (n = 69)
Age, median (range), years	48 (15‐81)
Sex, n	
Male	39
Female	30
Lesion characteristics, n	
Solitary	59
Multiple	10
Recurrent	2
Malignant transformation	2

### HPV Testing Results

HPV DNA was detected in 5 of 69 patients (7.2%). The screening test identified one high‐risk and four low‐risk HPV infections. Typing analysis revealed HPV‐6 in three cases, HPV‐11 in 1 case, and HPV‐16 in 1 case (Table 2).

**Table 2:** Characteristics of HPV‐Positive Pharyngeal Papillomas

Case	Age	Sex	Lesion type	Koilocytosis	Recurrent	HPV type
1	43	M	Solitary	+	Y	6
2	60	M	Solitary	+	N	11
3	23	F	Multiple	+	N	6
4	54	M	Multiple	+	Y	6
5	72	M	Solitary	+	N	16

### Tumor Size Distribution

Tumor sizes were distributed as follows: 1 to 4 mm (13 cases), 5 to 8 mm (9 cases), 9 to 12 mm (1 case), 13 to 16 mm (2 cases), 17 to 20 mm (0 cases), and >20 mm (2 cases). The 2 tumors larger than 20 mm corresponded to the 2 cases with malignant transformation.

### Malignant Transformation Cases

Two patients showed malignant transformation. Case 1 was HPV‐negative and involved multifocal lesions extending from the pyriform sinus to the posterior pharyngeal wall of the hypopharynx. The patient had undergone 2 prior resections at another institution, both diagnosed as squamous cell papilloma. Histopathology demonstrated papilloma with foci of well‐differentiated squamous cell carcinoma with focal stromal invasion. Tumor size was not documented in the pathology report; although the lesion grossly exceeded 20 mm at presentation, this case was excluded from tumor size analyses. Case 2 was HPV‐16 positive and arose from the anterior wall of the oropharynx (base of tongue). The specimen measured 25 mm. Histopathology showed marked papillary proliferation with focal stromal invasion consistent with squamous cell carcinoma.

### Koilocytosis

**Figure 1:**
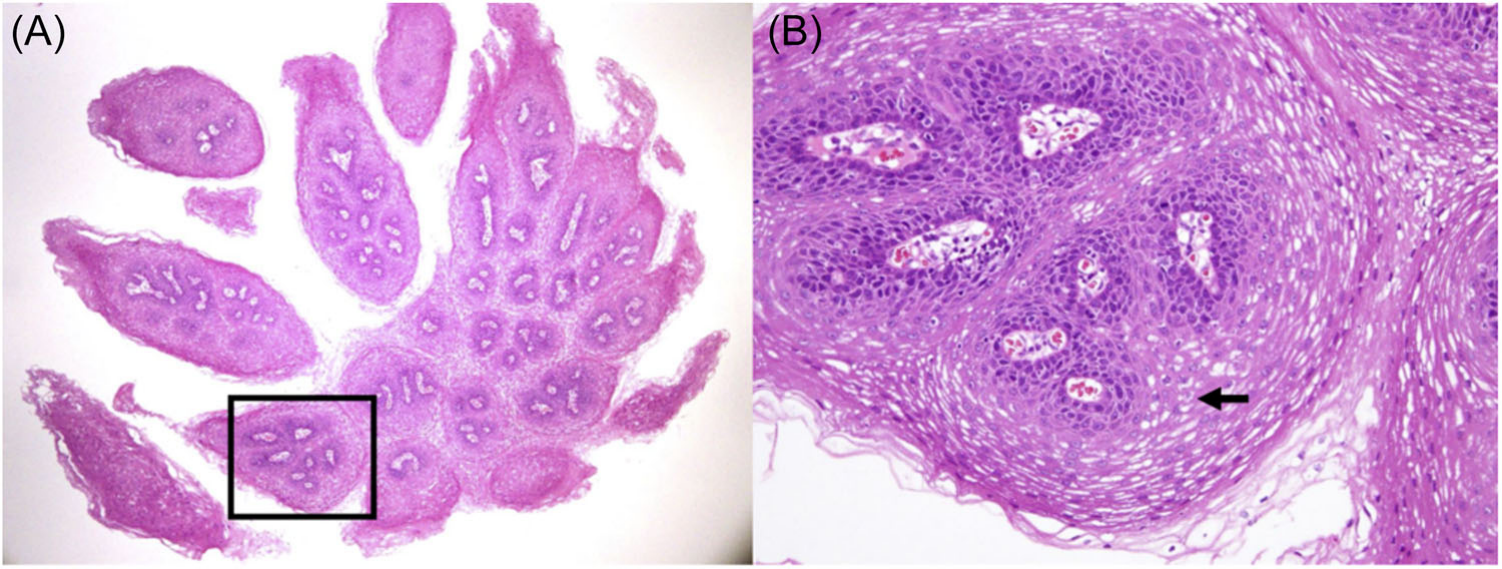
Pharyngeal squamous papilloma showing papillary proliferation and koilocytotic changes (arrows) with perinuclear clearing and nuclear enlargement (hematoxylin and eosin stain).

Koilocytosis was observed in 38 of 43 evaluated cases (88.4%), suggesting possible HPV involvement even in cases where HPV DNA was not detected. Representative histological features of koilocytosis are shown in Figure 1.

### Comparison of HPV‐Positive and HPV‐Negative Cases

The recurrence rate was significantly higher in HPV‐positive cases (40.0%) than in HPV‐negative cases (0%; *P* = .004). Although not statistically significant, HPV‐positive cases showed numerical trends toward higher rates of multiplicity (40.0% vs 12.5%) and malignant transformation (20.0% vs 1.6%). The average tumor size also tended to be larger in HPV‐positive cases (Table 3).

**Table 3:** Comparison of HPV‐Positive and HPV‐Negative Cases

Variable	HPV positive (n = 5)	HPV negative (n = 64)	*P* value
Recurrence, n (%)	2 (40.0)	0 (0)	.004*
Multiple lesions, n (%)	2 (40.0)	8 (12.5)	.150
Malignant transformation, n (%)	1 (20.0)	1 (1.6)	.141
Tumor size, mean ± SD, mm^a^	15.0 ± 10.0	5.8 ± 4.8	‐

^a^ Tumor size data available for 27 cases.

* *P* < .01.

## Discussion

Several studies have examined HPV prevalence in pharyngeal papillomas and reported widely varying detection rates. Our HPV detection rate of 7.2% was comparable to some reports but markedly different from others. For instance, Snietura et al reported 53.8% in Poland,^4^ Kim et al reported 25% in the United States,^5^ Donà et al reported 6.9% in Italy,^6^ and a previous Japanese study found 0%.^3^ This variability may be explained by regional differences and methodological diversity in HPV testing.

Regional variations in HPV infection rates have long been recognized. Tam et al conducted a meta‐analysis of 66 studies on oropharyngeal HPV infection in healthy individuals and reported an overall prevalence of 5.7%, with notable regional differences: the highest in South America (12.4%), followed by Europe (9.9%), North America (7.7%), and Asia (2.6%).^11^

Regarding testing methodology, no standardized HPV detection method has been established, and various assays have been used in previous studies. In this study, we used a commercially available test kit commonly employed in Japan for cervical cancer screening, which has been reported to have moderate analytical sensitivity.^12^ A notable finding of this study was the high prevalence of koilocytosis (88.4%) despite the relatively low HPV DNA detection rate (7.2%). This suggests that HPV infection may play a broader role in the pathogenesis of pharyngeal papilloma than DNA testing alone indicates. Koilocytosis reflects a cytopathic effect characteristic of HPV infection, and its frequent presence even in HPV‐negative cases may imply prior transient infection or viral clearance by host immunity. Pickard et al reported that during a three‐month follow‐up of oral HPV infection, only 39% of cases showed persistent infection,^13^ supporting the possibility of transient HPV infection in HPV‐negative papillomas. Therefore, histological evidence of koilocytosis may serve as an additional indicator of HPV involvement.

A key finding of this study was the absence of recurrence among HPV‐negative cases, in contrast to a recurrence rate of 40% in HPV‐positive cases (*P* = .004). Although the sample size of HPV‐positive cases was small, this difference suggests that HPV infection may be associated with a higher risk of recurrence in pharyngeal papillomas.

Tumors in HPV‐positive patients tended to be larger (15.0 ± 10.0 mm vs 5.8 ± 4.8 mm) and more frequently multifocal, suggesting greater proliferative potential. These findings support the notion that HPV infection contributes to more aggressive clinical behavior.

Recurrence in HPV‐positive cases may be explained by viral reactivation or reinfection triggered by mucosal injury after surgical excision. HPV infects basal epithelial cells through microabrasions, and surgical manipulation may create new sites susceptible to viral reentry. Matsuzaki et al reported that administration of the HPV vaccine can eliminate HPV DNA and reduce recurrence in patients with recurrent respiratory papillomatosis, a disease characterized by frequent recurrence and multiple lesions.^14^ This suggests that HPV vaccination might also have a preventive role in the recurrence of pharyngeal papillomas.

Clinically, our findings suggest several practical implications. HPV testing could help identify patients at higher risk of recurrence or malignant transformation, particularly those presenting with multiple or large lesions. Careful postoperative surveillance is warranted for HPV‐positive patients to enable early detection of relapse or malignant change. Future multicenter studies with larger cohorts and standardized HPV testing protocols are needed to validate these findings and clarify the clinical relevance of HPV infection in pharyngeal papillomas.

This study has several limitations. p16 immunohistochemistry was not performed. Although p16 is widely used as a surrogate marker of HPV‐driven biology in oropharyngeal squamous cell carcinoma,^15^ discordance between HPV DNA testing and p16 immunohistochemistry has been reported.^16^ Accordingly, the clinical and prognostic utility of p16 in benign papillomatous lesions remains uncertain. Because this cohort included cases dating back to 2006 and archived material was not uniformly available, systematic p16 evaluation was not feasible. Future studies integrating HPV testing with p16 immunohistochemistry in more recent, methodologically standardized specimens may clarify whether p16 provides complementary information for risk stratification and follow‐up planning.

The number of HPV‐positive cases was small, which limited the statistical power and generalizability of our findings. As this was a single‐center retrospective study conducted over more than a decade, institutional factors and changes in clinical practice may have influenced the results. The follow‐up duration varied widely, and some patients were referred back to primary physicians soon after surgery, potentially leading to underestimation of recurrence.

## Conclusions

In this retrospective study, HPV DNA was detected in 7.2% of pharyngeal papilloma cases.

HPV‐positive lesions were associated with higher rates of recurrence, multiplicity, and larger tumor size compared with HPV‐negative lesions, suggesting a more aggressive clinical behavior.

These findings indicate that HPV testing may provide useful information for assessing recurrence risk and guiding follow‐up strategies in patients with pharyngeal papilloma.

## Author Contributions

**Ryohei Asai**, conceptualization, data curation, formal analysis, writing—original draft; **Hiroumi Matsuzaki**, methodology, supervision, writing—review and editing; **Kiyoshi Makiyama**, supervision, validation, writing—review and editing; **Takeshi Oshima**, project administration, supervision, writing—review and editing.

## Disclosures

### Competing interests

None.

### Funding source

This work was supported by the Japan Society for the Promotion of Science KAKENHI [Grant Number JP24K12658].
